# HABS-BLOCKS© Reduced Harmful Algal Bloom Activity in an in-Lake Limno-Corral Mesocosm Case Study at Grand Lake St. Marys, Ohio (USA)

**DOI:** 10.1007/s00128-026-04228-9

**Published:** 2026-03-28

**Authors:** Stephen J. Vesper, Haley N. Hoehn, Morgan C. Grunden, Stephen J. Jacquemin

**Affiliations:** 1 United States Environmental Protection Agency, Cincinnati, OH 45268 USA; 2 Wright State University - Lake Campus, Celina, OH 45822 USA

**Keywords:** *Planktothrix*, Freshwater, Glucose, Cyanobacteria, Microcystin

## Abstract

HABS-BLOCKS© infused with glucose have been shown to reduce cyanobacterial populations and microcystin (MC) concentrations in laboratory mesocosm studies. An in situ limno-corral (LC) mesocosm study of HABS-BLOCKS© was conducted for two weeks during the 2025 algal bloom in Grand Lake St. Marys (GLSM) (Ohio, USA). Thirty HABS-BLOCKS© or Dummy HABS-BLOCKS© (contain no glucose) were added to three treated or three control LCs, respectively. Each LC holds approximately 1,000 L of lake water. After two weeks, cyanobacterial cell densities were 89% lower in treated compared to control LCs, and chlorophyll-a and phycocyanin concentrations averaged 80% and 77% lower, respectively. Initial MC concentrations averaged 41.4 µg/L, but treated LCs showed an average 89% reduction after two weeks. At the same time point, neither physical nor chemical parameters measured (except turbidity) were significantly different in the treated and control LCs. The treatment of GLSM water with HABS-BLOCKS© resulted in suppression of the *Planktothrix* population and decrease in MC concentrations. Further studies of HABS-BLOCKS© are needed to evaluate their applicability and safety.

## Introduction

The increasing frequency of harmful algal blooms (HABs) threatens freshwater ecosystems worldwide (Smucker et al. 2021). Harmful algal blooms drive eutrophication, can create a toxic environment, alter plankton biodiversity, and reduce the utilization potential of the water as a drinking water source for people and animals (Amorim and Moura 2021). Harmful algal blooms also hinder economic returns, which in Ohio have been shown to annually cause more than $150 million in damage (DeRose et al. 2021). Grand Lake St. Marys (GLSM) near Celina, Ohio, USA, has historically experienced one of the most persistent HAB problems in the USA (Jacquemin et al. 2018), which at its height in HAB activity ranked alongside Lake Apopka, FL as one of the most active blooms in public fresh waters (Clark et al. 2017).

For decades, the cyanobacterium *Planktothrix* has dominated the HAB in GLSM and is the primary source of the toxin microcystin (MC). While the biomass of the bloom tends to be associated with total phosphorus (TP) concentrations, MCs are associated with total nitrogen (TN) concentrations (Jacquemin et al. 2018, 2023). Microcystins are potent hepatotoxins because they have a strong affinity for serine/threonine protein phosphatases, which they inhibit (Campos and Vasconcelos 2010). In addition, MCs induce oxidative stress in animal cells (Campos and Vasconcelos 2010). Typically, the HAB in GLSM starts in the spring and persists through summer into fall. The median weekly microcystin concentration historically averages 23.2 µg/L (range 0.03–185.0 µg/L) (Jacquemin et al. 2023). The US EPA provides different microcystin thresholds for different water uses: for drinking water, a 10-day health advisory recommends levels at or below 0.3 µg/L for children under six and 1.6 µg/L for school-age children and adults**.** For recreational activities, the US EPA recommends a water quality criterion of 8 µg/L to trigger a swimming advisory (US EPA 2015). As a result of elevated MC concentrations in GLSM, the city of Celina, Ohio, which obtains water from the lake, is required to reduce toxin concentrations with expensive treatment technologies (Siddiquee et al. 2025).

The GLSM HAB occurs annually due to the inputs of nitrogen (N) and phosphorous (P) from its predominantly agricultural watershed. Efforts to mitigate nutrient levels with a variety of agricultural best management practices, legal requirements, and natural wetland habitat restoration have been highly successful in reducing the nutrients going into the lake (Jacquemin et al. 2018, 2023), but additional work, especially natural habitat restoration projects, is still needed to continue making progress (Jacquemin et al. 2025). In addition to challenges with external nutrient loading, there are legacy nutrients in the sediment that are difficult to remove which provide an internal load, particularly during certain times of year that can exacerbate environmental conditions (Jacquemin and Cubberley 2022). Despite some reduction in HABs severity recently because of nutrient load reductions, there is still a need for new HABs control technologies. These technologies will not solve the problem of excess nutrients moving into or cycling within the system but are needed to limit the impacts of HABs still occurring despite progress in nutrient reductions.

Presently, the addition of chemicals like hydrogen peroxide or copper sulfate are the most frequently used methods to control HABs in the USA. However, adding an oxidizer like hydrogen peroxide causes lysis of the cells and release of the toxins (Kim et al. 2024) and cyanobacterial populations soon return (Piel et al. 2024). Copper sulfate has been found to reduce cyanobacterial populations, but there are long-term, negative effects on non-target taxa, such as beneficial zooplankton (Watson et al. 2024). Non-toxic cyanobacterial control technologies are needed given the problems associated with existing methods.

Non-toxic technologies tested at GLSM have included nanobubble ozone technology (NBOT) (Chaffin et al. 2024) and various P binding products (Davidson et al. 2025). Nanobubble ozone technology was tested in mesocosms containing 2,000 L of GLSM water (Chaffin et al. 2024) wherein the ozone worked by oxidizing any organic matter in the water, including HABs. Overall, NBOT addition reduced HABs in GLSM water, but only for about 48 h. Reportedly, the ozone was consumed by the high cyanobacterial biomass and high dissolved organic carbon concentrations (Chaffin et al. 2024). Phosphorus binding treatments, like aluminum sulfate (alum) or lanthanum bentonite clay (Phoslock®), work by binding dissolved P and forming floccules that sink to the sediment. While these treatments yielded immediate decreases in chlorophyll and phycocyanin concentrations, reductions persisted for only about a week (Davidson et al. 2025). These short-term effects of P binding treatments are likely attributed to the rapid water exchange in the lake renewing the P (and subsequent bloom) in treated areas.

In our previous mesocosm studies, we reported that glucose suppressed the cyanobacteria, and the populations of heterotrophic bacteria, including Proteobacteria and Bacteriodetes, became dominant (Linz et al. 2024; Vesper et al. 2024). It appeared that the added glucose provided the heterotrophic bacteria with a competitive advantage over the cyanobacteria. Siddiquee et al. (2025) reported that before the *Planktothrix* bloom begins in GLSM, the heterotrophic Proteobacteria were the most abundant phylum (31.2%), followed by Bacteriodetes (23.6%), Actinobacteria (21.9%), and Verrucomicrobia (20.7%). As the lake temperature rises and the readily available carbon sources are depleted, the autotrophic cyanobacteria flourish. It may be that by adding glucose to GLSM water, the previously dominant heterotrophic bacteria might be allowed to regain their dominance. However, determining the exact mechanism that underpins the role of glucose in the GLSM microbial community is beyond the scope of this case study.

To provide glucose over the course of the bloom season, a floating, slow-release glucose source called HABS-BLOCKS© was created (Vesper et al. 2024). HABS-BLOCKS© are 2.5-cm cubes of pine, vacuum infused with glucose and covered with soy wax. HABS-BLOCKS© are intended to be a preventative measure and not a response to a bloom, compared to common control tactics like bentonite clay, hydrogen peroxide, copper sulfate, or ozone, which are typically applied based on bloom-level cell densities. In this study, HABS-BLOCKS© were tested in limno-corrals (LC) deployed in GLSM to determine if they could suppress the cyanobacterial populations and lower MC concentrations.

## Materials and Methods

### Description of Grand Lake St. Marys

Grand Lake St. Marys is a man-made reservoir originally constructed as a water source for the Miami & Erie Canal but now is used primarily as a drinking water source for the city of Celina, Ohio, and a public recreational resource. Grand Lake St. Marys is ∼52 km^2^ in surface area with an average depth of approximately 1.5 m (maximum depth 5 m). In the dock area where the study was conducted, the depth is a nearly uniform 2 m. The GLSM watershed is approximately 241 km^2^, dominated by row crops and livestock. Grand Lake St. Marys is in a temperate climate and experiences varying levels of winter ice cover (Newell et al. 2024).

### Production of Pine HABS-BLOCKS© or Dummy HABS-BLOCKS©

HABS-BLOCKS© were produced from 2.5-cm cubes of pine (PONGJA, Model: PJ-Cube-21, China). Glucose (Sigma-Aldrich, St. Louis, MO) was infused into the pine blocks by autoclaving them in a 4-L side-arm flask containing a saturated glucose solution (40 g glucose/80 mL of Milli-Q water). Air was removed and glucose infused into the pine blocks under vacuum. Blocks were then placed in a drying oven at 50 °C for two days to remove excess water. Dummy HABS-BLOCKS© are the same 2.5-cm cubes of pine but without infused glucose.

**Fig. 1:**
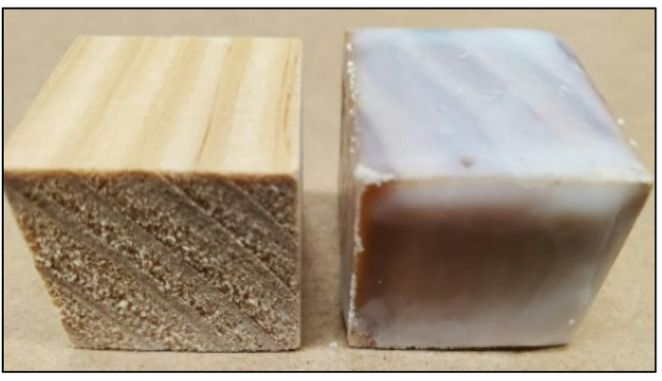
Pine blocks without soy wax covering (left) and with soy wax covering (right)

After drying the glucose infused blocks or the blocks without infused glucose, they can be covered with layer(s) of soy wax (Northern Lights Natural Soy Wax, Wellsville, NY) or left uncovered. To cover the blocks, soy wax was melted at 85°C and continuously mixed on a stir plate. Before applying soy wax, pine HABS-BLOCKS© were first placed at − 80 °C. (At this temperature, the layers of wax go on more uniformly). Then each cold pine block was covered by dipping the block into the melted soy wax. The HABS-BLOCKS© (Fig. 1) were then allowed to solidify in the refrigerator and a second layer added. Since HABS-BLOCKS© and Dummy HABS-BLOCKS© are fully biodegradable, they can be retrieved from the water’s surface after use and composted.

### Measuring Diffusion of Glucose from Pine HABS-BLOCKS© in Sterile Water

Studies of the dynamics of glucose diffusion from the HABS-BLOCKS© were conducted using glucose weight-matched HABS-BLOCKS©. HABS-BLOCKS© with either no soy wax covering or two layers of soy wax were added in duplicate to six sterile 250-mL beakers containing 100 mL of sterile Milli-Q water. These six beakers were then placed on a rotary shaker (New Brunswick Scientific, Edison, NJ) and mixed at 50 rpms. Every 24 h, the glucose concentration in each beaker was measured with Glucose Test Strips, (Precision Laboratories Inc, Cottonwood, AZ), following manufacturer’s directions. Then, each HABS-BLOCKS© and beaker was rinsed with two 100 mL aliquots of sterile water. After, 100 mL of sterile water and the HABS-BLOCKS© were returned to the beaker. The process was repeated daily until glucose was no longer detectable. (Glucose Test Strips’ lower detection limit is about 50 mg glucose/100 mL water.)

### Study of Cyanobacterial Control in Grand Lake St. Marys

**Fig. 2:**
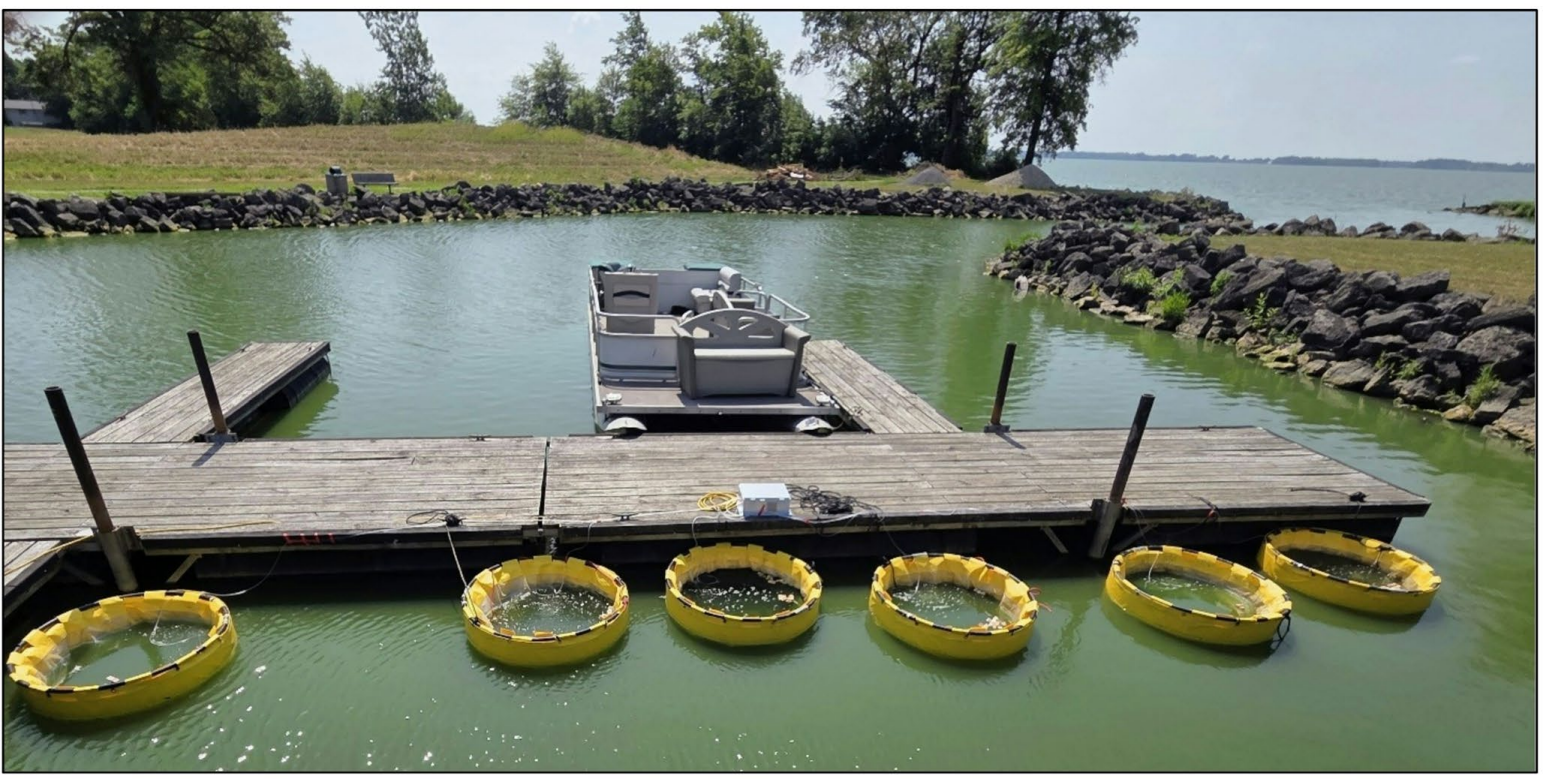
Limno-corrals as deployed, alternating treated and control

For this study, six cylindrical LCs were purchased (Curry Industries, Winnipeg, Manitoba, Canada) and deployed into GLSM at the Wright State University–Lake Campus’ dock on July 14, 2025 (Fig. 2). Each LC was 1-m in diameter and 2-m tall, containing approximately 1,000 L of lake water, open to the lake bottom and sealed there with a chain around the LC skirt. A long pole was used to press-down the skirt of each LC to ensure that each LC was fully extended to the bottom of the lake. Evidence that the LCs were sealed to the lake bottom was demonstrated by the muck covering the LC skirts at the time of the LC retrieval from the Lake.

### Treatment of LCs with HABS-BLOCKS© or Dummy HABS-BLOCKS©

Six LCs, three treated and three controls arranged alternately (Fig. 2), were deployed on July 14, and the water in the LCs was allowed to equilibrate overnight. On July 15, the initial 1 L water samples were collected from each LC. On July 15, three HABS-BLOCKS with two layers of soy wax or three “Dummy” HABS-BLOCKS©, each covered with two layers of soy wax, were added to each of the treated or control LCs, respectively. Then, six additional blocks were added on July 18 and on July 23. On July 25, fifteen more HABS-BLOCKS© or Dummy HABS-BLOCKS© were added for a total of 30. Each LC was aerated continuously using three Pond Air 2 75,000 aeration kits (Pond Air, St. Charles, IL, USA). Unfortunately, the aeration failed in Control LC2 on July 24 causing the water to become anoxic. Therefore, the July 29 data from LC2 was not used in statistical analyses.

**Fig. 3:**
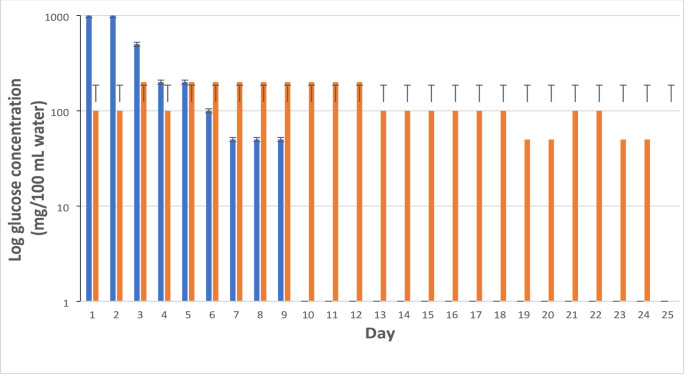
Log average daily glucose concentrations in 100 mL of sterile water exposed to HABS-BLOCKS© with either zero (blue) or two (orange) layers of soy wax. Each average and standard deviation is based on two replicate beakers each containing 100 mL of water (*N* = 2)

### *Planktothrix* Enumeration and Cyanobacterial Toxin Analysis

The *Planktothrix* concentration was enumerated using the Cellometer™ counting chamber (Nexcelom Bioscience, Lawerence, MA), following the manufacturer’s instructions. The *Planktothrix* concentration in GLSM was 1.4 × 10^4^ cells/mL on April 15, 2025. By June 24, the concentration was 6.1 × 10^4^ cells/mL, and on July 14, the *Planktothrix* concentration was 1.0 × 10^5^ cells/mL suggesting a bloom was amplifying and the experiment could begin.

Each week, 1 L of water was collected from a depth of 0.5 m in each LC and shipped overnight to the US EPA in Cincinnati, Ohio. Upon arrival, *Planktothrix* concentration was enumerated using the Cellometer™ counting chamber and replicate 5 mL aliquots from each 1 L LC sample were pipetted into 15-mL conical glass tubes (Pyrex, Corning, Corning, NY). These 5 mL aliquots were then frozen at − 20 °C until they were analyzed by EnviroSciences, Inc. (Stow, OH) for microcystin concentrations using enzyme-linked immunosorbent assay (ELISA), following US EPA Method 546.

### Measurement of Water Conditions

Each week, water quality parameters were measured in each LC, using a YSI proDSS Multiparameter Meter (Yellow Springs Instruments, Yellow Springs, OH). Replicate measurements made at a depth of 0.5 m included temperature, percent dissolved oxygen (DO%), dissolved oxygen concentration (DO mg/L), specific conductivity (SPC µS/cm), pH, and turbidity in formazin nephelometric units (FNU). (The DO probe was calibrated weekly, and the other probes were calibrated at the beginning of the experiment.) Concentrations of chlorophyll-a and phycocyanin were measured in replicate using an AlgaeTorch (bbe Moldaenke GmBh, Schwentinental, Germany). Glucose concentration in all LCs was measured weekly using Glucose Test Strips, following the manufacturer’s instructions.

### Statistical Analysis

Significance of differences in average measurements for each parameter measured in the treated and control LCs was evaluated using the Student T-test (T-test).

## Results

Pine blocks used in this study weighed on average 7.3 ± 0.4 g. After glucose infusion and drying, each block weighed on average 2.5 ± 0.7 g more. The addition of two layers of soy wax added an average of approximately 1 g to the weight to each block. Results of the glucose diffusion from HABS-BLOCKS© experiment are shown in Fig. 3. The HABS-BLOCKS© without a soy wax layer released their glucose in 9 days. The HABS-BLOCKS© with two layers of soy wax released their glucose in 25 days.

**Fig. 4:**
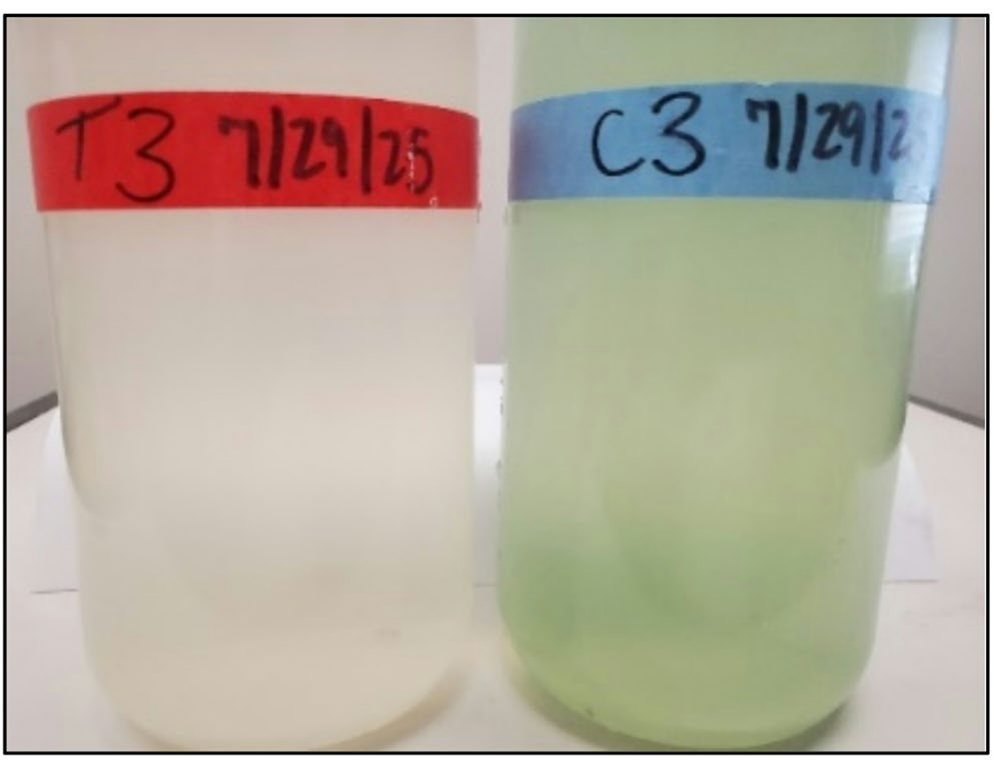
Water sample from the treated LC 3 (left) and the control LC 3 (right) on July 29, 2025

Table 1 shows that the *Planktothrix* concentration in the treated LCs averaged a 50% reduction after one week (though not statistically significant) and a significant (T-test,* p* = 0.003) 89% reduction after two weeks compared to the control LCs. Reductions in *Planktothrix* concentrations were reflected in reductions in chlorophyll-a and phycocyanin concentration each week of the experiment (Table 2). For example, after two weeks, the chlorophyll-a concentration was a significant (T-test, *p* = 0.01) 80% lower in the treated compared to the control LCs. Similarly, there was a significant (T-test, *p* = 0.01) 77% average reduction in phycocyanin concentration in the treated LCs resulting in an obvious visual difference in the water samples (Fig. 4).

**Table 1:** Average (AVG) ± standard deviation (SD) of *Planktothrix* concentration (Conc.) and AVG ± SD of microcystin concentration for each week in the Treated and Control limno-corrals (LC)

WEEK	0	1	2
Treated LCs AVG *Planktothrix* Conc. (cells/ml × 10^4^)	12.17 ± 2.5	5.08 ± 0.9	0.75 ± 0.7
Control LCs AVG *Planktothrix* Conc. (cells/ml × 10^4^)	11.33 ± 2.2	10.2 ± 4.4	7.10 ± 0.1*
T-test (Treated vs. Control) *p*-value	0.7	0.18	0.003
Treated LCs AVG Microcystin Conc. (µg/L water)	42.08 ± 1.9	35.25 ± 2.3	4.24 ± 2.8
Control LCs AVG Microcystin Conc. (µg/L water )	40.80 ± 0.2	51.40 ± 2.4	37.5 ± 6.1*
T-test (Treated vs. Control) *p*-value	0.40	0.01	0.007

Each average is based on three replicates (*N* = 3), except * was 2 replicates

**Table 2:** Average and Standard Deviation (AVG, SD) for YSI ProDSS Meter readings and AlgaeTorch measurements in the treated and control limno-corrals (with* p* values from T-tests noted) for each sampling week

Week	Control	Treated		Control	Treated		Control	Treated	
	0	0		1	1		2	2	
Measures	AVG; SD	AVG; SD	T-test	AVG; SD	AVG; SD	T-test	AVG; SD	AVG; SD	T-test
Temperature ° Celsius	29.8; 0.1	29.8; 0.1	1.00	26.4; 0.4	25.9; 0.2	0.14	28.1; 0	28; 0.1	0.4
Dissolved O_**2**_ % saturation	127; 9	130; 6	0.72	141; 20	115; 16	0.23	85; 5	81; 6	0.86
Dissolved O_**2**_ mg/L	9.6; 0.7	9.8; 0.4	0.7	11.3; 1.5	9.4; 1.3	0.23	6.61; 0.4	6.3; 0.4	0.66
Specific conductivity^**a**^	438; 8	441; 1	0.56	435; 7	436; 3	0.88	422; 2	413; 2	0.06
pH −log[H+]	8.4; 0.3	8.4; 0.1	0.73	8.7; 0.3	8.3; 0.2	0.11	7.9; 0	7.6; 0.1	0.1
Turbidity FNU^**b**^	59; 2	60; 1	0.93	54; 25	34; 5	0.32	34; 2	7; 3	**0.004**
Chlorophyll-a ug/L	469;17	478; 8	0.51	455; 3	409; 7	**0.001**	396; 22	80; 59	**0.01**
Phycocyanin ug/L	494; 29	511; 11	0.5	483; 2	444; 7	**0.002**	408; 25	94; 57	**0.01**

Each average is based on three replicates (*N* = 3), except for the control limno-corrals in week two, there were only 2 replicates. Statistically significant differences are bolded

^a^Microsiemens per centimeter (µS/cm)

^b^Formazin Nephelometric Units

At the start of the experiment, the initial MC concentration in GLSM averaged 41.4 µg/L of lake water. After one week, the average MC concentration was significantly (T-test,* p*-value = 0.01) reduced by 31% in the treated LCs and, after two weeks, by a significant (T-test, *p*-value = 0.007) 89% reduction in MC concentration in the treated LCs compared to the controls (Table 2).

Other water parameters measured, including temperature, DO% saturation, DO concentration, specific conductivity, and pH did not differ significantly in the treated and control LCs. However, as expected, DO% saturation and DO concentration trended lower in the treated LCs. Also, the average turbidity was significantly (T-test, *p* = 0.001) lower in the treated LCs compared to the control LCs after two weeks. Glucose was never measurable in the weekly test of the treated LCs.

## Discussion

Addition of HABS-BLOCKS© to GLSM water in the treated LCs resulted in significant reductions in the *Planktothrix* populations, chlorophyll-a and phycocyanin concentrations, turbidity, and MC concentrations. After two weeks, average microcystin concentration in the treated LCs was below the US EPA recreational activities advisory of 8 µg/L (US EPA 2015). These in-lake results are consistent with the results from our laboratory mesocosm studies (Vesper et al. 2022; Linz et al. 2024; Gastaldo and Vesper 2025). However, in-lake application of HABS-BLOCKS© presented unexpected challenges.

Reliance on wind and waves for aeration and mixing was not adequate in these small LCs. External aeration was needed to maintain oxygenation and mixing in the LCs. In larger LCs or in open water applications, external aeration may not be necessary.

Dosing with HABS-BLOCKS© was filled with many unknowns. Therefore, only a few HABS-BLOCKS© were added initially. Dosing was increased based on the field data for adequate DO and on-site observations of water color and clarity. Monitoring the glucose concentrations weekly was not useful, since the glucose disappeared quickly. Glucose monitoring may require daily or even hourly testing after the HABS-BLOCKS© are first added to the LCs.

Another problem encountered was not knowing the optimum time to begin HABS-BLOCKS© treatment. Unlike clay, hydrogen peroxide, copper sulfate, or ozone, which are added when the bloom appears, HABS-BLOCKS© are designed to prevent HAB development by adding them before the bloom amplifies. By July 15, the date of HABS-BLOCKS© introduction, the bloom had accelerated as the water temperature reached 29.8 °C. Huang et al. (2025) determined that water temperature was a major driver of HABs development. Therefore, in addition to monitoring *Planktothrix* concentrations, close monitoring of water temperature may help predict bloom development in GLSM. Proper timing for HABS-BLOCKS© addition should improve their use efficiency. Finding effective methods to predict harmful algal blooms in lakes is an active area of research (Park et al. 2024; Schaeffer et al. 2024; Zahir et al. 2024).

Because HABs are occurring more frequently and developing in lakes where they have never occurred before (Fang et al. 2022; Sahu et al. 2025), less toxic approaches to HABs control are needed. Some of the currently utilized chemicals, like hydrogen peroxide and copper salts, have toxicity and environmental concerns, but new formulations may help (Sinha et al. 2018). Numerous new approaches evaluated for HABs control include photocatalytic oxidation, nano-bubble enabled foam fraction, iron (Fe) precipitation of P, synthetic non-metallic nanoparticles (NMNPs), and others. However, these technologies rely on complex chemical formulations and modalities.

Photocatalytic oxidation, although less energy demanding than some other technologies, relies on a photocatalyst fabricated of molybdenum disulfide nanosheets with bismuth oxyhalide (Xu et al. 2026). According to the authors, more research will be needed to determine their economic and environmental applicability. Nano-bubble enabled foam fraction to remove cyanobacteria and toxins relies on surfactants, including cetyltrimethylammonium bromide and sodium dodecyl sulfate (Zhang et al. 2026). Laboratory studies demonstrated effectiveness, but tests in real lake water suggested that the complex water matrixes (e.g., salinity and dissolved organic matters) affected foaming ability and reduced target algal removal rates. Iron additions in many forms have been tested to control HABs over the past couple of decades (Aubriot et al. 2025). The Fe causes P precipitation, flocculation and sinking of the cyanobacteria. However, Fe application is sensitive to redox conditions. After treatment with Fe, the cyanobacteria population returned in a matter of days to weeks (Aubriot et al. 2025). Synthetic NMNPs such as carbon nanotubes, carbon nanodots, and cell-penetrating peptides have been explored to treat HABs (Antrim et al. 2025). Acute toxicity was reported to be cyanobacterium strain specific. The authors concluded that different NMNP types might be useful in treating specific cyanobacteria but are not generalizable. It is also not clear if these materials are cost effective or environmentally neutral.

Of course, any HAB control or treatment technology is not the long-term solution. The long-term solution to HABs is the reduction in nutrient loading of rivers and lakes (Paerl et al. 2018). Restoration of wetlands around GLSM has begun, but nutrients have been added to GLSM for generations, and it will take years to correct the problem (Jacquemin et al. 2023). Unfortunately, climate change may make progress even more difficult. Zhang et al. (2025) reported that, for China’s lakes and reservoirs, climate change may already be off setting the progress made from the nutrient control efforts. Nevertheless, long term progress involving sustainable nutrient reduction approaches must carry future efforts. But, in the short term, there is a need for safe and effective HAB control or treatment methods. The addition of glucose in the form of HABS-BLOCKS© covered with two layers of soy wax may be one such method. However, this feasibility study is only the first step in a long process to determine the practical applicability of HABS-BLOCKS©. There are many additional factors for inclusion in future studies, including the evaluation of potential health and safety concerns and possible short- and long-term ecosystem impacts.

## Data Availability

All data will be available at the NIH-PMC website.

## Declarations

### Generative AI in Scientific Writing

Statement: No generative AI was used in the scientific writing of this manuscript.
